# GO@β-Ag_2_MoO_4_ Composite: One-Step Synthesis, Characterization, and Photocatalytic Performance against RhB Dye

**DOI:** 10.1021/acsphyschemau.4c00038

**Published:** 2024-09-10

**Authors:** Pedro Hyug de Almeida da Silva, Dalete Araújo de Souza, Rubens Lucas de Freitas Filho, Ana Paula de Carvalho Teixeira, Rochel Montero Lago, Walter Ricardo Brito, Edgar Alves Araújo Junior, Litiko Lopes Takeno, Francimauro Sousa Morais, José Fábio de Lima Nascimento, Yurimiler Leyet Ruiz, Libertalamar Brilhalva Saraiva, Francisco Xavier Nobre

**Affiliations:** † Departamento de Química, Instituto Federal de Educação Ciência e Tecnologia do Amazonas (IFAM), Campus Manaus Centro, 69020-120 Manaus, AM, Brazil; ‡ Departamento de Química, ICEX, Universidade Federal de Minas Gerais, 31270-901 Belo Horizonte, MG, Brazil; § LABEL – Laboratory of Bioelectronic and Electroanalytic, Central Analytical Lab, Federal University of Amazonas (UFAM), 69077-000 Manaus, AM, Brazil; ∥ Laboratório Interdisciplinar de Materiais Avançados (LIMAV), Universidade Federal do Piauí (UFPI), 64049-550 Teresina, PI, Brazil; ⊥ Departamento Acadêmico de Infraestrutura – DAINFRA, Instituto Federal de Educação Ciência e Tecnologia do Amazonas (IFAM), Campus Manaus Centro, 69020-120 Manaus, AM, Brazil; # Laboratório de Ensaios Mecânicos, Automação e Simulação (LEMAS), Polo de Inovação (INOVA), Instituto Federal de Educação Ciência e Tecnologia do Amazonas (IFAM), 69075-351 Manaus, AM, Brazil; ∇ Laboratório de Processamento de Materiais (LPMat), Centro de Ciências Exatas, Universidade Federal do Amazonas, 69077-000 Manaus, AM, Brazil

**Keywords:** photocatalysis, composite, silver molybdate, semiconductors, Rietveld refinement, RhB dye

## Abstract

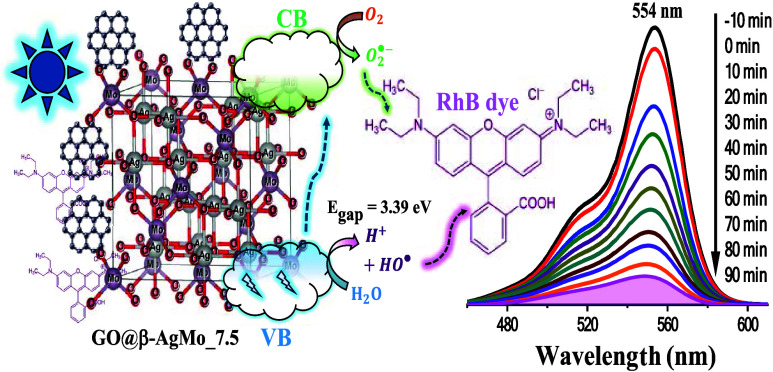

The combination of materials to improve properties of interest has become one of the strategies widely used for numerous applications, including new catalysts, over the last few decades. In this study, silver molybdate (β-Ag_2_MoO_4_) microcrystals were efficiently obtained by the hydrothermal method, obtaining composites with different amounts of graphene oxide (GO) (1, 2.5, 5, 7.5, and 10%, w/w) using the conventional hydrothermal method. The incorporation of GO on silver molybdate was confirmed by X-ray diffraction (XRD) and Raman spectroscopy, where the vibrational modes and crystallographic planes characteristic of the materials of interest were highlighted. The images collected by scanning electron microscopy (SEM) revealed the occurrence of plate-shaped structures (shells) anchored to the surface of the silver molybdate microcrystals (core). The optical properties showed that the materials presented *E*_gap_ between 3.34 and 3.39 eV, where the sample with 7.5% of GO (GO@β-AgMo_7.5) was the one that presented energy for the conduction band, largely favorable to the formation of superoxide radicals through the photoexcitation process of electrons. The catalytic tests demonstrated that, among the samples obtained in this study, the sample with 7.5% of GO (GO@β-AgMo_7.5) exhibits superior photocatalytic performance against the dye rhodamine B (RhB) in an aqueous medium. Thus, the kinetics constant for photolysis (absence of catalysts) and for the sample β-AgMo and the sample with 7.5% of GO (GO@β-AgMo_7.5) are 0.38 × 10^–3^, 12 × 10^–3^, and 23.72 × 10^–3^ min^–1^, respectively. Therefore, it is 62.5 times more efficient in the degradation of the RhB dye, which confirms the promising photocatalytic properties of the obtained composite.

## 1 Introduction

Climate change, mainly associated with human intervention, has compromised the maintenance of ecosystems, especially concerning the quality of water resources essential for the survival of species.^1^ Currently, the textile industry has been one of the most responsible sectors for the contamination of water bodies, although it is also directly or indirectly related to atmospheric and land pollution.^2^ Therefore, it is notable that this sector needs special attention to deal with the impacts it has on the environment.^3^

During the dyeing process of natural or textile fibers, around 10–15% of the dyes used are released into wastewater, therefore requiring specific treatment to remediate the contaminants and secondary pollutants present, being considered a potential environmental risk.^4^ Therefore, these compounds cause a decrease in biochemical dissolved oxygen (BOD) and reduced transmittance of natural sunlight; consequently, the decrease in the natural photosynthetic process^5^ occurs.

Textile dyes pose a high risk to aquatic organisms, as they are toxic, mutagenic, and bioaccumulation, and they also cause complications to human health, particularly in the liver, spleen, and nervous system.^6^ Aiming to reduce environmental impacts and meet established government standards, several treatment methods have been developed and applied in the textile industries, which differ in cost, the volume of effluent to be remediated, and nature of the effluent to be treated.^7^ Among the conventional methods, it is possible to highlight physical or chemical adsorption,^8^ physical–chemical processes,^9^ microbiological,^10^ and advanced oxidative processes—AOPs.^11^

AOPs consist of a technology widely used in wastewater treatment and industrial effluents due to its high capacity to degrade and/or discolor organic compounds, in addition to its low selectivity.^12^ The method basically consists of the formation of radicals with high oxidizing potential, which act in the partial or total transformation of a high-molecular-weight compound into a simpler compound or a substance with low or no relative toxicity.^11,13,14^ Several techniques include AOPs; in this sense, it is possible to highlight the ozone treatment,^15^ sonochemical,^16^ electrochemical,^17^ and photochemical processes.^18,19^ The photocatalytic process is commonly initiated by the absorption of light by semiconductors (photocatalysts) that accelerate the photochemical process, which can be classified into homogeneous and heterogeneous catalysts.^20^ In the first case, the catalyst is in the same physical state as the reaction medium. Conversely, in heterogeneous photocatalysis, the catalyst is in a different physical state, generally the solid state. Heterogeneous catalysts are more versatile due to the ease of removing the reaction medium after the reaction process and the possibility of reuse in several cycles.^16,20,21^

Heterogeneous photocatalysis is based on the activation of a semiconductor by natural or artificial sunlight in an aqueous medium.^22^ The absorption of photons promotes electrons from the valence band of the semiconductor to the conduction band (BC), thus producing holes (h^+^) in the valence band (BV), allowing the formation of radicals with high oxidizing power, such as hydroxyl radicals (OH^•^) and superoxide radicals (O_2_^•–^), which are capable of degrading a finite variety of organic compounds.^17,20,22^ It is worth mentioning that the promotion of electrons from the valence band to the conduction band is only possible when the absorbed energy is equal to or greater than the bandgap energy (*E*_gap_) of the semiconductor.^12^

Among the semiconductors used for this purpose are titanium oxide—TiO_2,_^23^ silver phosphate—Ag_3_PO_4_,^24^ metal sulfides (CuS, ZnS, MoS, e CdS),^25^ polymorphs of silver tungstates—Ag_2_WO_4_,^26^ and silver molybdates—Ag_2_MoO_4_.^21^^21^ Silver molybdate is a well-known semiconductor that has attracted the attention of researchers due to its multiple applications due to its antibacterial properties,^27^ lubricant,^28^ photocatalyst^14^^14^ and photoluminescent.^29^

The crystallization of Ag_2_MoO_4_ leads to polymorphic forms, in this case, tetragonal (α-phase) and cubic (β-phase).^30^ The literature reveals that the α-Ag_2_MoO_4_ phase is thermodynamically metastable, undergoing phase transition to the β-Ag_2_MoO_4_ phase at temperatures above 280 °C.^31^ Although the β phase of silver molybdate is the stable phase among the polymorphs displayed for the structures formed, this compound suffers a reduction in photocatalytic properties over several cycles due to the oxidation–reduction process of the silver ions present in the structure. Thus, approaches are required to circumvent this process. In this context, obtaining heterojunctions with other materials allows for better photon absorption performance and improvements in the stability of the materials obtained.

Recent studies have presented the excellent properties of graphene as a support material for obtaining heterojunctions, namely, in the study carried out by Padmanabhan et al.^32^ A systematic literature review reinforces graphene oxide’s benefits when coupled with titanium dioxide. This combination contributes to stability in photochemical processes and increases the adsorptive, semiconducting, and capacitive properties. On the other hand, Lewandowski et al.^33^ report the heterojunction obtained by combining graphene oxide and silver phosphate, acquiring excellent photocatalytic properties and stability after 4 consecutive photocatalytic cycles in the photodegradation of phenol in an aqueous medium.

Given the above, the present study reports the obtaining and characterization of β-Ag_2_MoO_4_@graphene by the hydrothermal method, characterized by different analytical techniques and used as a photocatalyst in the degradation of rhodamine B (RhB) dye in an aqueous medium under ultraviolet radiation.

## 2 Materials and Methods

### 2.1 Synthesis of β-Ag_2_MoO_4_ and GO@β-Ag_2_MoO_4_

The synthesis of silver molybdate was carried out according to the steps described by Souza et al.^34^ Initially, 2 mmol of silver nitrate—AgNO_3_ (Sigma-Aldrich, purity >99.9%) and 1 mmol of sodium molybdate dihydrate—Na_2_MoO_4_·2H_2_O (Sigma-Aldrich, purity >99.9%) were solubilized in 40 mL of distilled water, separately, mixed with the aid of Vortex stirring. After the salts were totally solubilized, the solution containing the molybdate ions (MoO_4_^2–^) was transferred to a hydrothermal reactor with a Teflon cup, which remained under constant magnetic stirring.

After the salts were completely solubilized, the solution containing the molybdate ions (MoO_4_^2–^) was transferred to a hydrothermal reactor with a Teflon cup and remained under constant magnetic stirring. Then, the solution containing silver ions (Ag^+^) was slowly added to the solution containing the MoO_4_^2–^ using a Pasteur pipette until complete transfer, which remained under constant and vigorous magnetic stirring for 1 min.

At the end of the period described, the system was closed and placed in an oven for hydrothermal processing for 2 h at 120 °C. Then, the system was cooled and collected by centrifugation (4000 rpm for 3 min) and washed several times with distilled water to remove expectant ions. The precipitate was dried in a circulating air oven at 100 °C for 24 h; this sample was identified as β-AgMo.

Following the same methodology adopted in the synthesis of the β-AgMo sample, the composites were obtained using the hydrothermal method, therefore, differing only by adding the masses of 1, 2.5, 5, 7.5, and 10% of graphene oxide GO (Sigma-Aldrich, purity >99.9%) in relation to the mass of β-AgMo (w/w), obtaining the samples: GO@β-AgMo_1, GO@β-AgMo_2.5, GO@β-AgMo_5, GO@β-AgMo_7.5, and GO@β-AgMo_10, respectively. In this case, a fixed mass of 2 g of silver molybdate was adopted, and from this, the masses of graphene oxide were obtained, acquiring the relation. Thus, for each of the proportions presented, the respective mass of graphene was introduced into the solution containing the MoO_4_^2–^ ions, which remained under constant magnetic stirring. In contrast, the solution containing the Ag^+^ ions was added slowly until complete transfer of this solution. The system was closed and subjected to hydrothermal treatment at 120 °C for 2 h in an oven and subsequently cooled at the end of the synthesis process. Finally, it was collected using centrifugation (4000 rpm for 3 min) and washed several times with distilled water to remove spectator ions, followed by drying in a circulating air oven for 24 h at 100 °C.

### 2.2 Characterization

The powders obtained were structurally characterized by X-ray diffraction (XRD) using the Shimadzu equipment, XRD 7000, applying radiation from a copper anode (Cu Kα = 0.154056 nm), adopting the powder method. Crystallographic information was collected in the 2θ range between 5° and 100°, with a step of 0.02°. Raman vibrational information was collected by operating the BWTek Raman spectrometer, model i-Raman Plus, using a laser with an excitation wavelength of 532 nm. For all samples, the spectra were collected in the range of 50–4000 cm^–1^, using 85% of the total laser power (10 mW) and 10 coadditions. The morphology of the materials and the semiquantitative analysis of the elements present in the matrix were investigated using field emission scanning electron microscopy (SEM-FEG), using the Tescan equipment, Vega 3. The samples were placed in aluminum stubs, and carbon ribbon was used without the need for gold vapor metallization by sputtering. The optical bandgap energy (*E*_gap_) was determined through UV–vis spectroscopy using diffuse reflectance (UV–vis/DRS). The UV–vis/DRS spectrum was acquired by the Shimadzu UV-2600 UV–vis spectrophotometer, collecting the reflectance (*R*%) in the range from 200 to 1000 nm, where barium sulfate (BaSO_4_) was used as the internal reflectance standard.

### 2.3 Photocatalytic Performance over RhB Dye

The photocatalytic performance of the synthesized materials was investigated by using rhodamine b (RhB) dye molecules. Initially, 50 mL of solution at a concentration of 10 mg L^–1^ of the RhB dye was added to a reactor with 20 mg of the catalyst under constant magnetic stirring without electromagnetic radiation. The obtained suspension was stirred with an ultrasonic washer for 10 min in the absence of light at room temperature to obtain adsorption equilibrium. Then, a 0.5 mL aliquot was removed, and the suspension was exposed to ultraviolet radiation (UVc) from six Philips germicide lamps (15 W each, 90 W total output power) with a wavelength of 253.7 nm. The photocatalytic experiments were performed for 90 min, thus removing 0.5 mL aliquots at consecutive intervals of 10 min. The suspension collected at each interval was centrifuged for 5 min at 10,000 rpm, the supernatant was collected, and the spectrum of the solution was measured in the range of 200–900 nm, observing the absorbance of the solution at 554 nm.

## 3 Results and Discussion

### 3.1 X-ray Diffraction Pattern and Structural Rietveld Refinement

**Figure 1:**
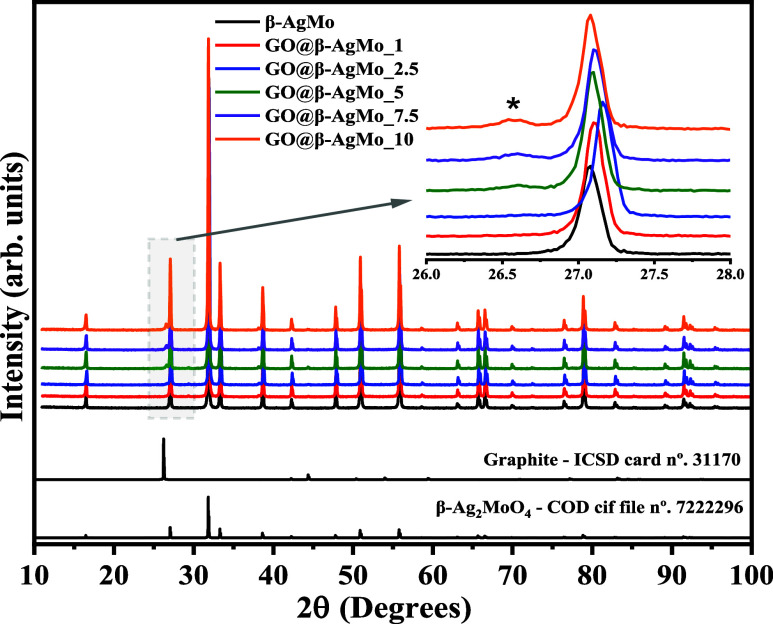
X-ray diffraction pattern of silver molybdate and the composites. The theoretical XRD patterns of graphite and Ag_2_MoO_4_ were collected from ICSD card No. 31170 and COD. cif file No. 19318, respectively.

Figure 1 presents the diffraction patterns collected for pure silver molybdate (β-AgMo) and the composites GO@β-AgMo_1, GO@β-AgMo-2.5, GO@β-AgMo_5, GO@β-AgMo_7.5, and GO@β-AgMo_10. Furthermore, the theoretical diffraction pattern was obtained from the Inorganic Chemistry Structure Database (ICSD) card No. 31170^35^ and Crystallography Open Database—COD. cif file No. 7222296,^36^ referring to the graphite structure and silver molybdate’s β phase, respectively.

The diffraction pattern collected for β-AgMo revealed the presence of peaks with intensity and profile that indicate obtaining a material with a high degree of crystallinity and short and long-range ordering.^37−40^ Furthermore, no peaks appeared associated with the presence of secondary phases or remnants of synthesis precursors. Therefore, confirming the obtaining of a pure phase for silver molybdate, the success of the proposed synthesis for the material of interest is confirmed. Indexing the β-AgMo diffraction pattern resulted in high similarity with the crystallographic information in the COD. cif file No. 7222296, which has a cubic structure and space group point group of *Fd*3̅*m* and *O*_*h*_^7^, respectively. All characteristic crystallographic planes in the 2θ intervals between 10 and 100° are characteristics of the β phase.

Obtaining the composites was confirmed by the presence of the diffraction peak in 2θ = 26.5°, associated with the crystallographic plane (002̅), characteristic of carbon-derived structures.^41−44^ In this case, the crystallographic information about the graphite on the ICSD No. 31170 card was used, which also agreed with studies reported in the literature.^45,46^ Therefore, the gradual increase of the mentioned diffraction peak was confirmed with the increase in the amount of graphene in the composites’ composition, as shown in the inset of Figure 1, in the 2θ range from 26 to 28°.

In order to study the crystallographic information on the materials obtained in detail, we decided to conduct a structural refinement, adopting the Rietveld method.^47−50^ For these purposes, FullProf software,^51^ August 2023 version for Windows, was used, adjusting the profile and intensity of diffraction peaks by computing experimental data (*Y*_obs_) and theoretical (*Y*_cal_) data using the pseudo-Voigt Thompson-Cox-Hastings function, axial divergence asymmetry. The quality of the calculated data was monitored by the value of the quality parameters *R* (*R*_e_, *R*_p_, *R*_wp_, *e*, χ^2^) as well as the difference contained in the residual line (*Y*_obs_ – *Y*_cal_) when computing theoretical and experimental values. It is important to highlight that the theoretical data for silver molybdate and graphene oxide, used in structural refinement, were extracted from the card ICSD No. 31170 and COD. cif file No. 19318, respectively.

**Figure 2:**
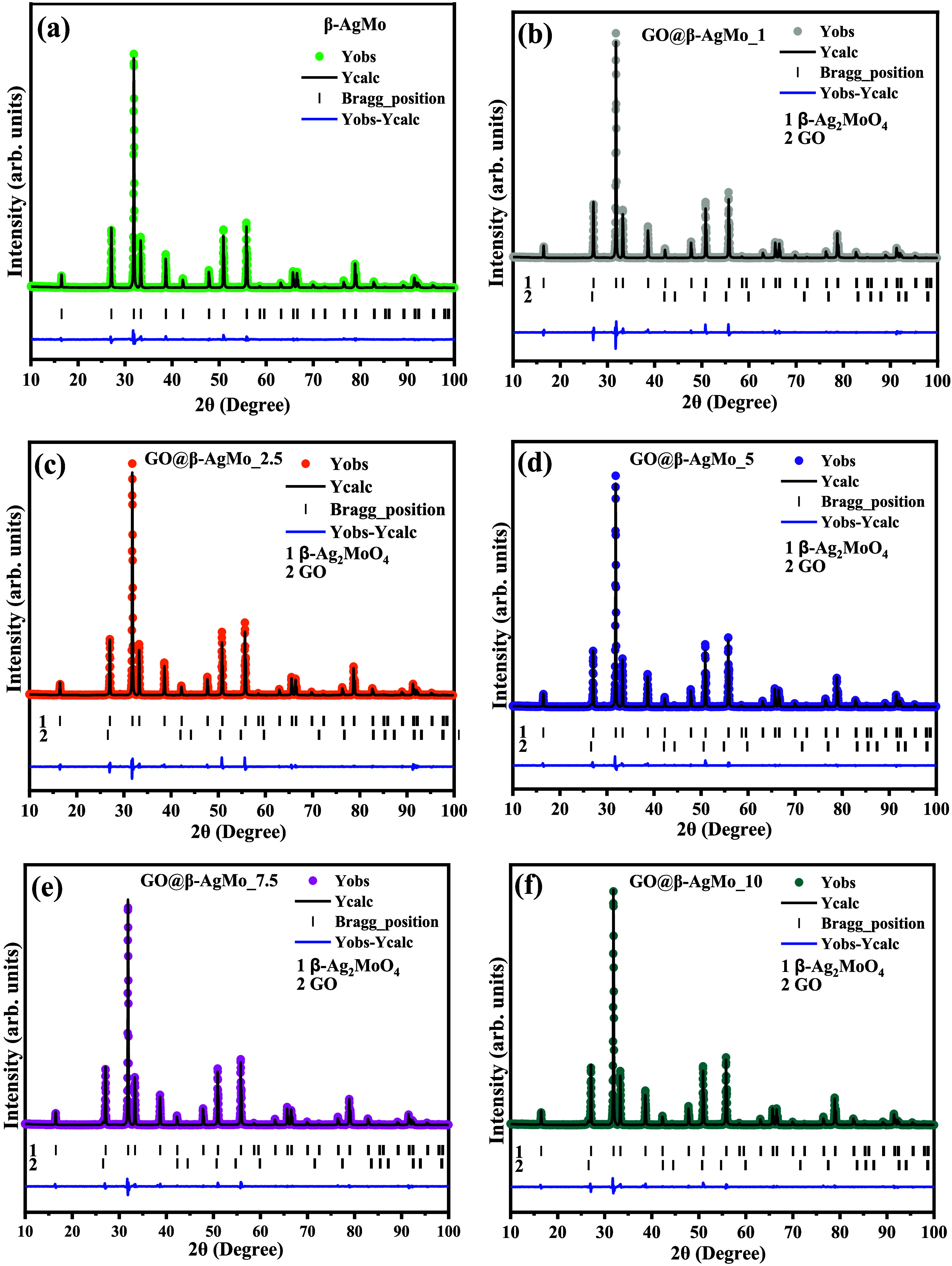
Structural Rietveld refinement plot for (a) bare silver molybdate and (b–f) heterojunctions.

In Figure 2a–f, the plots obtained for the structural refinement with all of the samples obtained are presented, where the Bragg peaks, represented by 1 and 2, are associated with the position of the crystallographic planes of silver molybdate and graphene oxide. In this case, it has a diffraction pattern that is highly similar to the diffraction pattern of graphite. Therefore, in Figure 2, it is possible to verify the excellent agreement of the experimental and theoretical profile for all of the samples studied, indicated by the residual line presented, confirming the high purity of the silver molybdate obtained in the present synthesis route as well as the presence of Bragg peaks characteristic of graphene oxide, which makes up composites.

From the crystallographic study, through structural refinement using the Rietveld method, it was possible to investigate the behavior of the lattice parameters; in this case, the cubic system has exhibited *a* = *b* = *c*, and unit cell volume (V), which depend on the synthesis conditions and/or other conditions adopted in sample processing. As shown in Figure S1, the size of the lattice parameters, represented by “*a,”* and the unit cell volume (*V*), had similar behavior, where the size of the crystallographic axis of the unit cell for pure silver molybdate was *a* = 9.314(1) Å, which was increased by the addition of graphene oxide percentages in the proportions of 1 and 2.5%, obtaining *a* = 9.332(6) Å. However, there was a decrease for additions of 5, 7.5, and 10% (m/m) graphene oxide, which reached *a* = 9.3140 Å (GO@β-AgMo_10). For bare silver molybdate, the unit cell volume behaved analogously, where *V* = 808.02(6) Å^3^ was obtained for the sample β-AgMo; there is then an expansion of the volume when the percentages of 1 and 2.5% until you reach V = 812.84(6) Å^3^, then gradually reduce the volume until reaching *V* = 807.99(1) Å^3^, in this case, for the sample GO@β-AgMo_10.

The observed behavior may be related to the crystal-directing effect of graphene oxide nanoplates in the reaction medium. Consequently, it enabled particle-oriented growth due to the point charges distributed on the surface of the graphene oxide nano- and microplates. In the study reported by Ahamad and Ahmed,^52^ which adopted structural refinement in the study of composites containing zinc oxide and graphene oxide, crystallographic variations were also noted, associated with the occurrence of crystalline defects and oxygen vacancies.

The contributions of microstrain and crystallite size on the width at half height of the diffraction peaks were investigated by adopting the model proposed by Williamson–Hall.^53,54^ By plotting the β_Total_ cos θ vs 4 sin θ, it is possible to adjust the data using linear fit so that the angular coefficient of the straight line obtained after the adjustment is equivalent to micro deformation. At the same time, the intercept is equivalent to *k*·λ/*D*_*hkl*_. Therefore, it is possible to obtain the crystallite size.

In Figures S1 and S2 and Table 1, the crystallite size and micro deformation are summarized; in addition, the values of lattice parameters and unit cell volume are obtained through the structural refinement of the synthesized and reported samples in the literature.

**Table 1:** Rietveld Refinement Results for Lattice Parameters (*a* = *b* = *c* and *V*), Crystallite Size (*D*_*hkl*_), and Microstrain (ε) Obtained in This Study and Reported in the Literature

	lattice parameters				
sample	*a* (Å)	*V* (Å^3^)	*D*_*hkl*_ (nm)	ε × 10^–5^	reference
β-AgMo	9.314(1)	808.02(6)	79.8	8.40	this work
GO@AgMo_1	9.328(3)	811.71(1)	74	–5.94	this work
GO@AgMo_2.5	9.332(6)	812.84(6)	71.8	–17.4	this work
GO@AgMo___5	9.316(1)	808.48(7)	73.8	–18.6	this work
GO@AgMo_7.5	9.314(1)	808.02(6)	77.2	–8.09	this work
GO@AgMo_10	9.314(0)	807.99(1)	77.5	–8.03	this work
	9.318(5)	809.01(7)			(28)
	9.3177(4)	808.96(6)			(36)

As seen in Table 1 and Figure S2, the highest slope value of the straight line obtained to fit the data in the Williamson–Hall plot was obtained for the pure silver molybdate sample, indicating the highest microstrain value in this case, ε = 8.10 × 10^–5^, and crystallite size equals 79.8 nm. Furthermore, it was confirmed that the smallest crystallite size was obtained for the sample containing 2.5% graphene in its composition, i.e., sample GO@β-AgMo_2.5, which exhibits micro deformation ε = −17.4 × 10^–5^. It is important to note that except for the β-AgMo sample, all others exhibit a negative sign for the angular coefficient and microstrain. This is due to the noncorrelation of crystallite size with the microstrain, which can vary anisotropically due to the contribution of graphene nanoplates or microplates in the reaction medium, which influenced the decrease in crystallite size up to a concentration of 2.5%.

However, the lowest microstrain value was obtained for the GO@β-AgMo_5 sample, which increased the crystallite size and microstrain for the following concentrations. Furthermore, the values obtained for the lattice parameters agree with those reported in the literature consulted.^28,36^

Table S1 summarizes the values of atomic positions (*x*, *y*, and *z*), isotropic thermal factor (B_iso_), and occupancy factor (O_cc_) obtained from structural refinement for all refined samples. In this case, variations, mainly related to the occupancy factor and atomic coordinates, can be noticed, indicating the variation in the length of the bonds due to the short-range movement of the atoms within the unit cell, therefore confirming the structural changes caused in the silver molybdate structure forming in situ in the presence of graphene oxide in different proportions.

**Figure 3:**
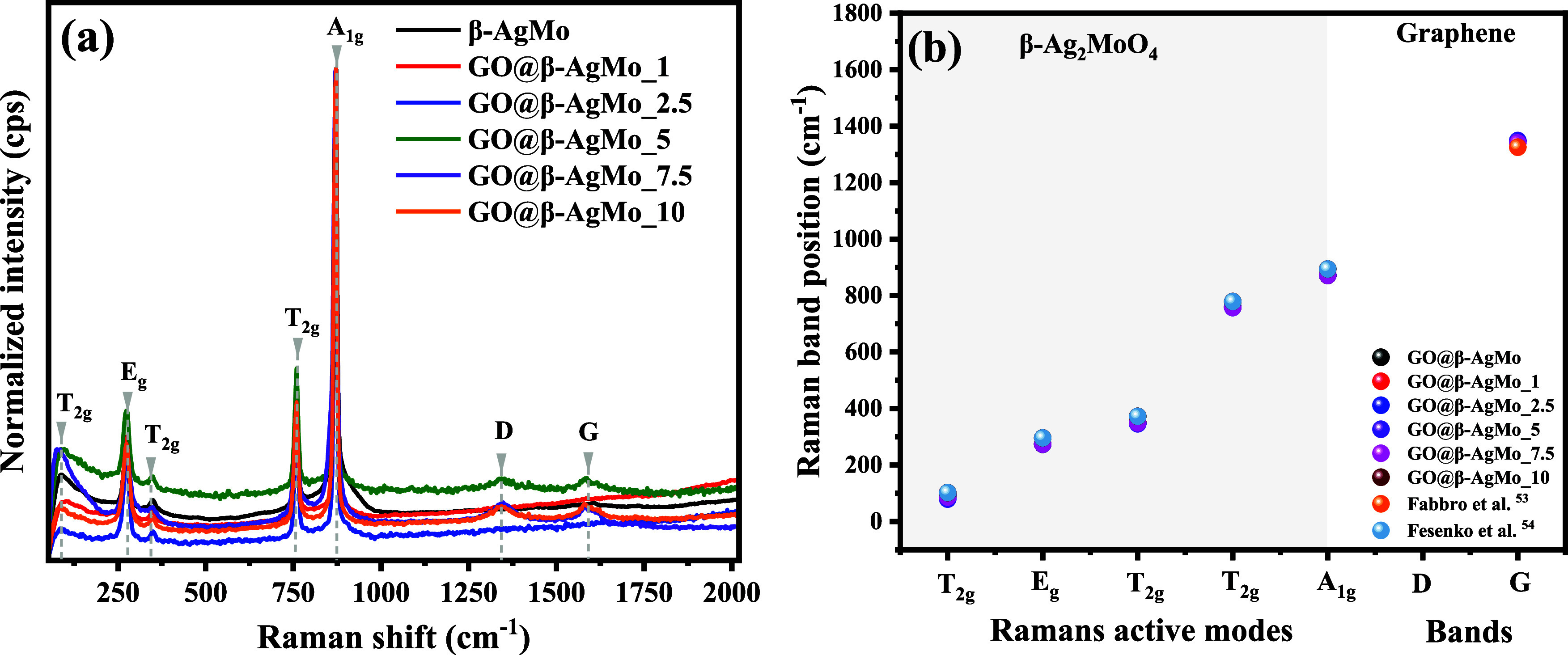
Vibrational Raman spectrum of (a) silver molybdate and composites and (b) Raman band positions against Raman active modes of synthesized samples and reported in the literature.

Vibrational characterization is a powerful tool in material characterization. It enables the correlation between information obtained by X-ray diffraction and other structural analyses. In this study, we chose to investigate the vibrational modes of materials synthesized by Raman spectroscopy, as seen in parts of Figure 3a,b.

Group theory reveals that the cubic-spinel structure of silver molybdate exhibits five active modes in Raman spectroscopy, represented by the irreducible formula: Γ_Raman_ = A_1g_ + E_g_+ 3T_2g_. Therefore, in the Raman spectrum presented in Figure 3a, it is possible to clearly identify the five vibrational modes characteristic of the silver molybdate structure between 50 and 2000 cm^–1^. The strong intensity band associated with active vibrational mode A_1g_, at 870 cm^–1^, is related to the symmetric stretching of the Mo–O bonds present in [MoO_4_] clusters with tetrahedral geometry.

The asymmetric stretching of Mo–O bonds is associated with the mode T_2g_ identified by the presence of the band at 756 cm^–1^, as well as at 352 and 88 cm^–1^, above all, with lower relative intensity compared to the band identified at 756 cm^–1^, due to the contributions of ion movements of Ag^+^ in the crystal lattice. Finally, the mode located at 277 cm^–1^ (E_g_) is the doubly degenerate mode related to the vibrations of the Ag–O bonds in the [AgO_6_] clusters with octahedral symmetry.

The Raman vibrational spectrum for the composites revealed the appearance of two bands, most prominent in the samples containing the percentages of 5, 7.5, and 10%, that is, the samples GO@β-AgMoO_5, GO@β-AgMo_7.5 e GO@β-AgMo_10, associated with the D and G bands, characteristic of the monolayer structure of graphene oxide.^44^ Furthermore, an increase in the intensity of the respective bands is noted with an increase in the proportion between graphene oxide and silver molybdate, corroborating the information presented in X-ray diffraction.

Figure 3b and Table S2 present the plot of the band position vs the vibrational modes for pure silver molybdate and composites. The results also correlated with the band position of the active modes reported in the literature. Based on the results presented, it is clearly noticeable that the positions for the bands associated with the active modes agree with those reported by Fabbro et al.,^55^ and Fesenko et al.^56^

**Figure 4:**
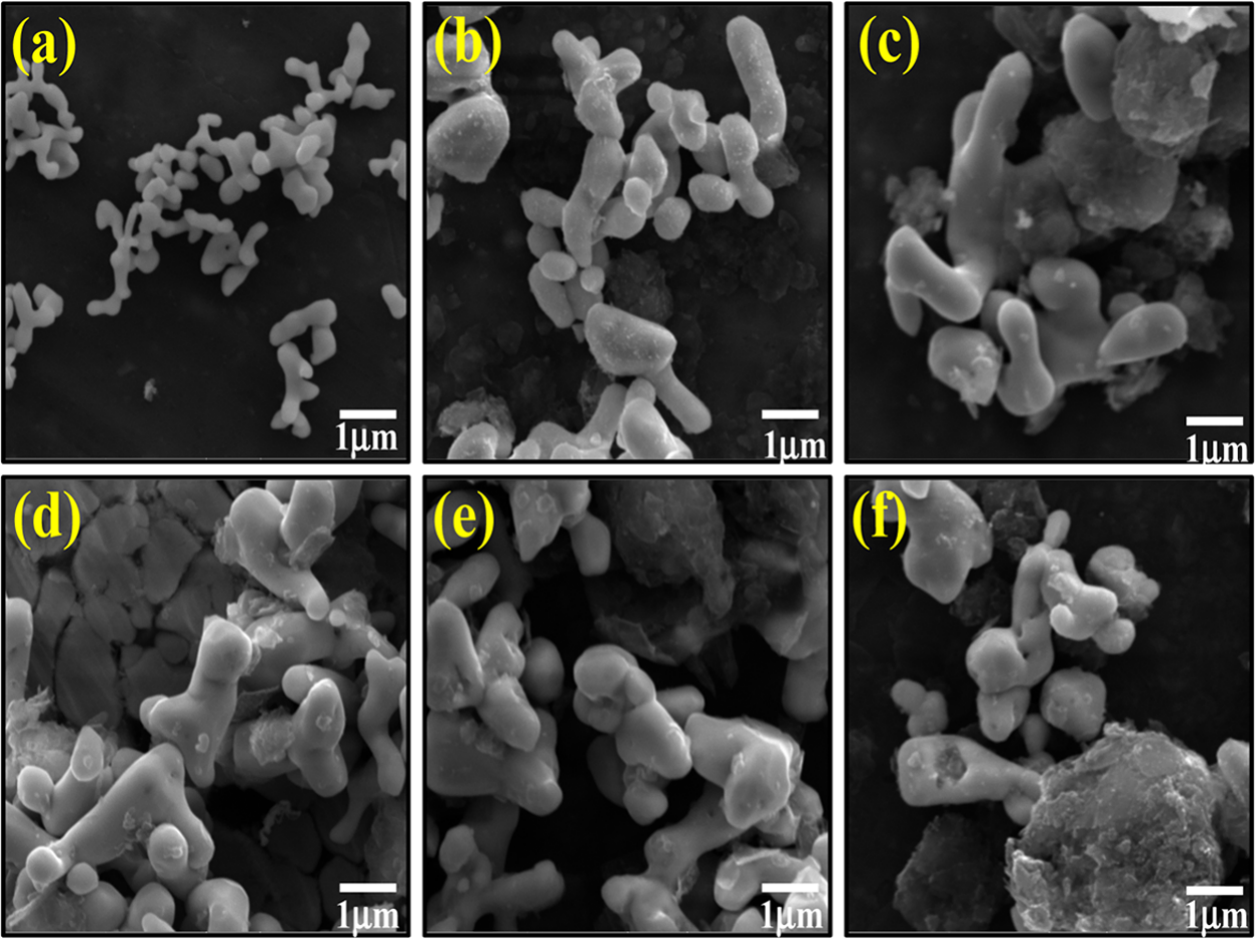
Scanning electronic microscopy images of (a) β-AgMo, (b) GO@β-AgMo_1, (c) GO@β-AgMo_2.5, (d) GO@β-AgMo_5, (e) GO@β-AgMo_5, and (f) GO@β-AgMo_10.

The morphological and semiquantitative analysis of silver molybdate and heterojunctions was carried out using scanning electron microscopy (SEM), as shown in parts of Figures 4a–f, S3, and S4.

Based on the image presented in Figure 4a, referring to the β-AgMo sample, that is, bare silver molybdate, it is possible to notice the occurrence of microcrystals with a morphology similar to corals, in agreement with the literature consulted.^37^ This morphology for silver molybdate is commonly reported when the microcrystals are obtained using the hydrothermal or solvothermal method.^34,37,57^ It suffers from variations in morphology when surfactant compounds or solvents with a high nonpolar character are added. In the study reported by Cunha et al.,^36^ silver molybdate microcrystals were efficiently obtained using the hydrothermal method, with the addition of different alcohols in the reaction medium. Therefore, potato-shaped morphologies, corals, and elongated rods were obtained as well as a reduction in crystal size when the polarity of the solution was increased.

In Figure S3, the histogram obtained for the length of silver molybdate microcrystals is available, in this case, collecting the length of 100 microcrystals from the SEM images of the synthesized materials. Therefore, it is possible to note that bare silver molybdate presented 92% of microcrystals with an average size between 0.5 and 2.5 μm. As for the heterojunctions, it is noted that microcrystals were grown with the addition of graphene oxide in relation to pure silver molybdate. However, it did not follow a linear behavior. Furthermore, it is noted that there was a decrease in homogeneity for the size ranges. Therefore, for the samples GO@β-AgMo_1, GO@β-AgMo_2.5, GO@β-AgMo_5, GO@β-AgMo_5, and GO@β-AgMo_10, the intervals with the greatest crystal length were, respectively, 1 and 4 μm (88%), 1 and 6 μm (95%), 1 and 5 μm (88%), 1 and 7 μm (98%), and 1–5 μm (93%). This effect can be related to the arrangement of the graphene oxide sheets that support the nucleation and growth of microcrystals, influenced by the effect of surface charges resulting from the groups present on the graphene oxide surface.

Although evidence of heterojunction formation was easily observed from samples containing 5% graphene oxide in the mixture (GO@β-AgMo_5) in Raman spectroscopy and X-ray diffraction, it is possible to notice from the images collected by SEM that particles with nano- and microscale dimensions, characteristic of graphene oxide, are seen in Figure 4b–f, for all prepared heterojunctions. Furthermore, it is suggested that small GO particles may have been trapped within the silver molybdate microstructures, thus justifying the variations observed for the increase in volume and unit cell network parameters up to a concentration of 5%, as presented in the X-ray diffraction technique.

The energy-dispersive X-ray spectrum (EDS) of the samples obtained is shown in Figure S4. Based on the results obtained, it is possible to note that pure silver molybdate has only dispersive energy peaks associated with the elements molybdenum, silver, and oxygen, in the proportion 1:2:6.5, respectively.

Therefore, an excess of oxygen is observed, which may be related to the oxygen atoms coordinated with the silver and molybdenum atoms on the surface of the microcrystals. On the other hand, for samples containing different proportions of graphene oxide, there was the appearance of a dispersive energy peak at 0.27 keV, characteristic of the carbon element, in this case, derived from graphene oxide, therefore confirming the presence of graphene oxide for all heterojunctions obtained. This result confirms that in proportions lower than 5% of graphene oxide in the composition of the heterojunctions, although there was no evidence of relative intensity for the diffraction plane characteristic of graphene oxide, as well as the molecular vibrations associated with the D and G bands, in Raman spectroscopy, it is possible to verify, in the images and semiquantitative analysis by EDS, that the characteristic elements of graphene oxide are present. Furthermore, variations were confirmed between the atomic percentages of molybdenum and oxygen and molybdenum carbon due to the increased amount of graphene oxide in the heterostructures. The ratio between silver and molybdenum were 2.10, 2.03, 2.03, 2.4, and 2.3 for the samples GO@-AgMo_1, GO@-AgMo_2.5, GO@-AgMo_5, GO@-AgMo_7.5, and GO@β-AgMo_10, respectively. On the other hand, following the same order between samples, the ratio between carbon and molybdenum was 3.10, 9.93, 14.6, 22.5, and 23.4.

The optical properties of the synthesized materials were studied by spectroscopy in the ultraviolet and visible regions using diffuse reflectance (UV–vis/DRS).^58^ Initially, percentage diffuse reflectance (*R*%) spectra were collected as a function of wavelength (λ) in the spectral range between 190 and 800 nm. This data was converted into energy per photon (*E*_pho_) using Planck’s equation (*E*_pho_ = 1240/λ), while the percentage reflectance data were converted into the Tauc function.^59^ In this study, the value of E_gap_ was obtained by extrapolating the paraboloid curve obtained from the plot of (*αE*_pho_)^*n*^ against *E*_pho_, adopting directly permitted transitions, i.e., *n* = 2, in agreement with the studies carried out in the literature.^34,57^

**Figure 5:**
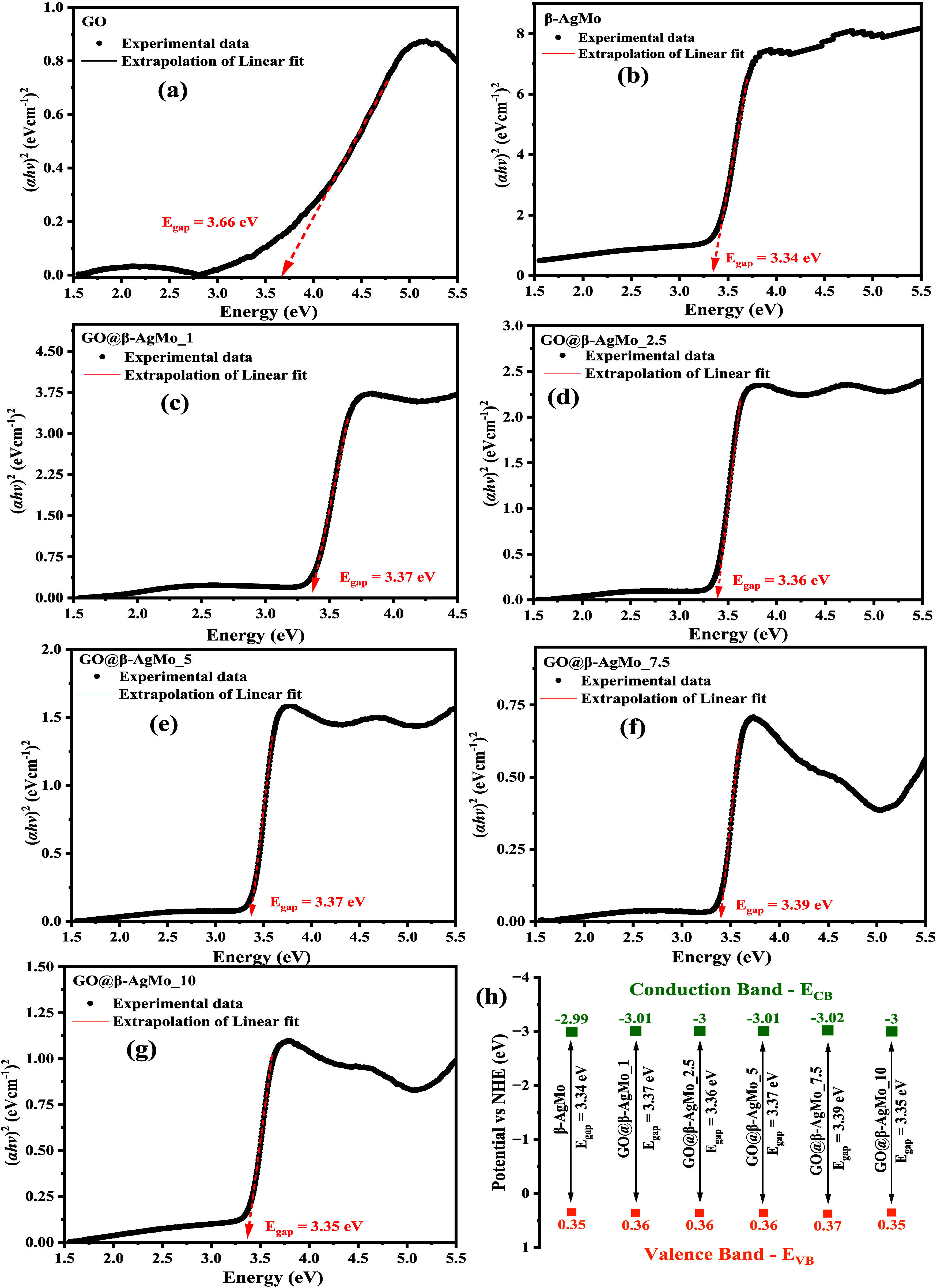
UV–vis spectra by diffuse reflectance (DRS) of (a) GO, (b) β-AgMo, (c) GO@β-AgMo_1, (d) GO@β-AgMo_2.5, (e) GO@β-AgMo_5, (f) GO@β-AgMo_7.5, (g) GO@β-AgMo_10, and (h) energy of valence and conduction band of samples.

In Figure 5a–h, the Tauc plots for graphene oxide, silver molybdate, and heterojunctions are presented with their respective bandgap energy values obtained by extrapolation of the paraboloid curve, as well as the position energy value of the bands valence (*E*_VB_) and conduction band (*E*_BC_).

Based on the results obtained, it is possible to see in Figure 5a that the *E*_gap_ value of graphene oxide was 3.66 eV, associated with electronic transitions of the type π → π* and n → π*, involved between the orbitals of the carbon elements (C sp^2^ and sp^3^) and oxygen (O 2p) from different groups present on the surface of graphene oxide structures. According to the literature,^60^ the *E*_gap_ of carbon-rich materials is directly related to the morphology, particle size, and method of obtaining different structures. In the study carried out by Méndez-Romero et al.,^61^ bandgap values close to 3.1 eV were obtained, reducing this value to close to 1.1 eV when functionalized with octadecylamine. On the other hand, in the study reported by Lundie et al.,^62^ calculations using the ab initio method and hybrid functional density (DFT) report a bandgap value between 3.66 and 3.88 eV.

The E_gap_ value for silver molybdate was 3.34 eV (Figure 5b), characteristic of semiconductors that absorb photons in the ultraviolet spectral region (UV). Particularly, they involve electronic transitions between orbitals Ag 4d and O 2p, located in the valence band (VB), for the orbitals Mo 4d and O 2p, located in the conduction band (CB).^55^ These electronic transitions can be influenced by structural and morphological characteristics, mainly associated with the synthesis methods and conditions adopted. Fabbro et al.^55^ report the variation in E_gap_ for silver molybdates synthesized by the chemical precipitation method. It indicates changes in structural, morphological, and optical properties when adopting solvent, distilled water, ethanol, and ammonia, resulting in values of E_gap_ of 3.32, 3.33, and 3.29 eV, respectively.

The obtained *E*_gap_ for composites GO@β-AgMo_1, GO@β-AgMo_2.5, GO@β-AgMo_5, GO@β-AgMo_7.5, and GO@-AgMo_10, as available in Figure 5c–e, are 3.37, 3.36, 3.37, 3.39, and 3.35 eV, respectively. These results reveal that there was an increase in the E_gap_ value after the combination of silver molybdate with GO due to the contribution of the optical properties of GO, which has a higher *E*_gap_ compared to β-AgMo. In this way, it is possible to confirm that there was, in fact, no chemical doping of the structure to the point of introducing intermediate levels between the bands (BV and BC), indicating the formation of a heterojunction due to the physical mixing of the materials, corroborating what was observed in the characterization by scanning electron microscopy.

In the study by Naghani et al.,^63^ by obtaining zinc oxide nanocomposites with graphene oxide, the combination of the materials increased the *E*_gap_ from 3.37 to 3.63 eV, which, according to the authors, is due to the increase in excitation energy due to the reduction in particle size.

From the *E*_gap_ values obtained by the Tauc method, using diffuse reflectance UV–vis spectroscopy, it was possible to investigate the contribution of the formation of heterostructures to the energy associated with the valence band (*E*_VB_) and conduction band (*E*_CB_) of silver molybdate. In Figure 5h, the plot for the energy of the bands is presented for the samples β-AgMo, GO@β-AgMo_1, GO@β-AgMo_2.5, GO@β-AgMo_5, GO@β-AgMo_7.5, and GO@β-AgMo_10. The *E*_CB_ and *E*_VB_ values were calculated using eqs 1 and 2 in accordance with the literature consulted.^12,29,61^
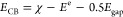
1
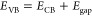
2where *E*^e^ is the energy of the free electron (*E*^e^ = 4.5 eV) and χ is the electronegativity of β-Ag_2_MoO_4_, calculated from eq 3, where χ_Ag_, χ_Mo_ and χ_O_ are 4.44, 3.9, and 7.54 eV, respectively.
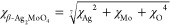
3

According to the results obtained, as shown in Figure 5h, it is possible to observe that the *E*_VB_ and *E*_CB_ values obtained for pure silver molybdate (β-AgMo) were 0.35 and −2.99 eV, respectively, in agreement with that reported in the literature.^13,21^ These band energy values are sufficient to promote the oxidation of water molecules, decomposing them into species H^+^ (1.23 V vs NHE) and HO^–^ (1.99 V vs NHE), as also, the formation of radicals HO^•^ (2.40 V vs NHE), through the holes (h^*+*^) located in BV. On the other hand, electrons excited to the conduction band provide the opportunity for the formation of superoxide radicals—O_2_^•–^ (−0.28 V vs NHE), hydroperoxide HO_2_^•^ and hydrogen peroxide – H_2_O_2_ (1.44 V vs NHE), by the reduction of oxygen molecules dissolved in an aqueous medium.^64^ These results confirm the electron-donating behavior of β-Ag_2_MoO_4_, agreeing with the literature consulted,^65^ as an n-type semiconductor.

However, small variations were noted for the positions of the *E*_VB_ and *E*_CB_ bands for the heterojunctions, GO@β-AgMo_1, GO@β-AgMo_2.5, GO@β-AgMo_5, GO@β-AgMo_7.5, and GO@β-AgMo_10. Among these samples, composite GO@β-AgMo_7.5 stands out, which presented the highest *E*_VB_ and *E*_CB_ values, respectively, 0.37 and −3.02 eV. Based on these results, it is possible to indicate that the generation of radicals with oxidizing and reducing potential by this material occurs more easily compared to other samples obtained since the higher the *E*_CB_ and *E*_VB_ value, the more favorable the oxidative processes in the medium aqueous by the absorption of photons by the semiconductor.^66^

The photocatalytic performance of bare silver molybdate and heterojunctions was investigated through the photodegradation of rhodamine B (RhB) dye molecules in an aqueous medium, as seen in parts of Figure S5. For these purposes, except for photolysis, i.e., the absence of a catalyst in the photodegradation experiment, the RhB dye solution was used at a concentration of 10 mg L^–1^ and a catalyst dosage of 2 g L^–1^, exposed to 90 min of radiation UVc with a photon energy of 4.8 eV under an aeration rate of 1.8 Lmin^–1^ and constant magnetic stirring.

The RhB dye exhibits maximum absorption at wavelength 554 nm, which displays a strong pink color, characteristic of the chromophore group present, which belongs to the class of compounds called xanthenes.^67^ In this way, the cleavage and subsequent breaking of the bonds present in the dye chain led to discoloration or mineralization of the solution, with a decrease in the characteristic maximum absorbance, depending on the time of exposure to radiation. Based on the information graphically presented, it is possible to note that the exposure of the RhB dye solution to UVc radiation was insufficient to completely discolor the simulated effluent, with around 79% of the initial concentration remaining after 90 min of exposure.

On the other hand, when silver molybdate microcrystals, as well as heterojunctions, were added as catalysts for the photodegradation reaction of the RhB dye molecules, it was possible to notice an evident decrease in the maximum absorption of the dye at the wavelength of 554 nm, a result of the breaking of bonds in the chromophore group of the dye. In this way, the percentages of discoloration are 58, 62, 73, 81, 85, and 90%, regarding catalysts GO@-AgMo_1, GO@-AgMo_2.5, GO@-AgMo_5, GO@-AgMo_10, and GO@β-AgMo_7.5, respectively. Furthermore, it is noted that pure GO was completely adsorptive, in this case, corresponding to 60% adsorption after a period of 10 min of equilibration of the suspension in the dark under ultrasonic agitation and 85% after 120 min under exposure to UVc light.

Based on these results, the GO@AgMo_7.5 sample presented the best photocatalytic performance compared to the other samples obtained, thus causing a synergistic effect for the photocatalytic process in the presence of RhB dye molecules.

**Figure 6:**
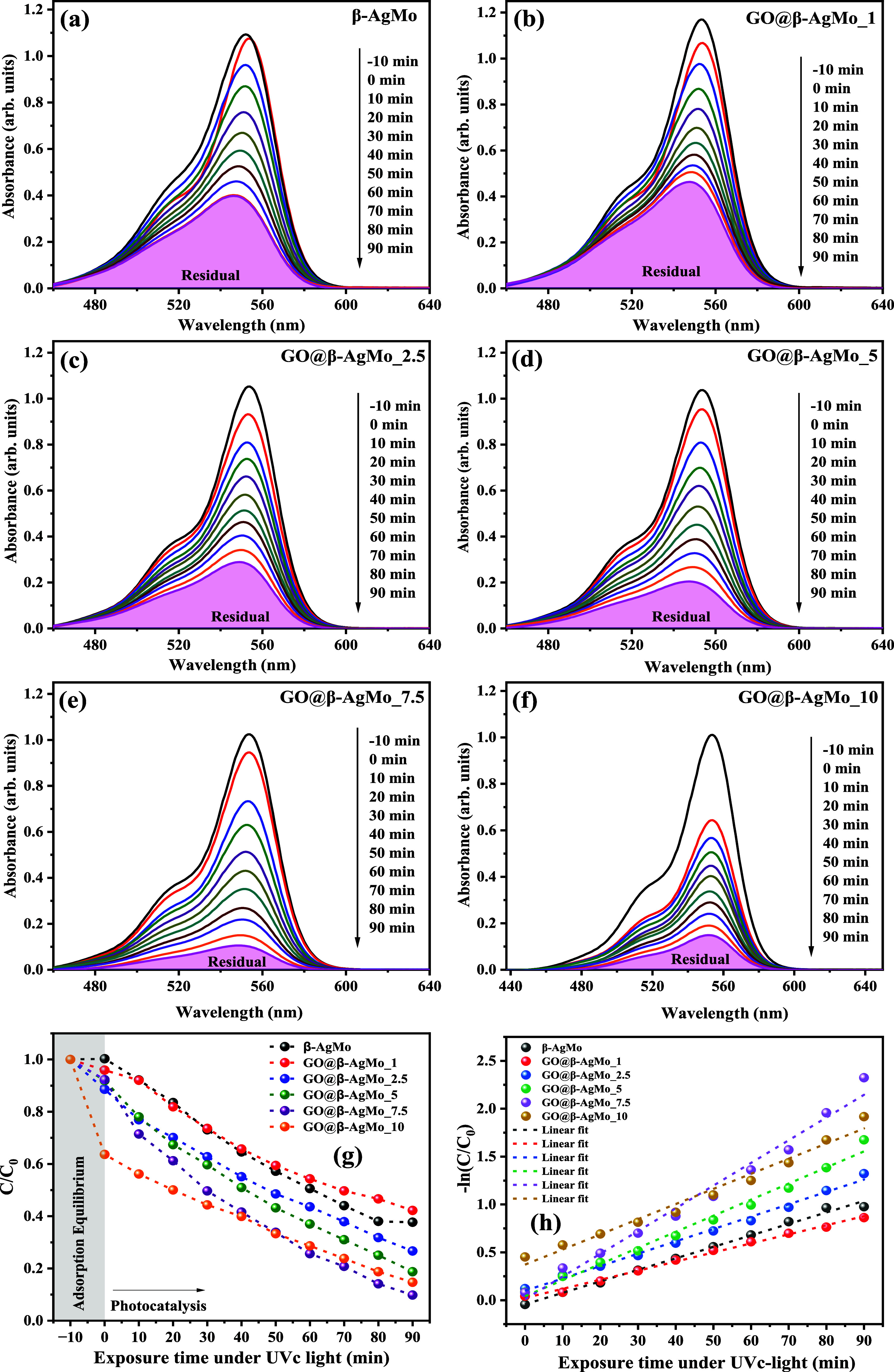
UV–vis spectrum of RhB dye solution against exposure time under UVc light in the presence and absence of bare Ag_2_MoO_4_ and graphene oxide and silver molybdate heterojunctions.

The evolution of the decolorization process of RhB dye solutions was studied by plotting *C*/*C*_0_ as a function of the exposure time to UVc radiation, where *C* is the absorbance associated with a given time *t* and *C*_0_ is the absorbance of the initial concentration of the RhB dye, as shown in Figure 6a–h. Therefore, it is possible to observe that the graphic profile obtained for all photocatalytic tests carried out is characteristic of pseudo-first order reactions, except for the GO sample, which presented an adsorptive profile that did not follow a photodegradation kinetic profile.
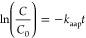
4

The apparent rate constant, obtained from the slope of the linear adjustment of the data presented through the plot of −ln(*C*/*C*_0_) vs exposure time (Figure 6g,h) and eq 4, resulted in the value of 0.38 × 10^–3^ min^–1^ for the test carried out in the absence of catalyst, that is, photolysis. On the other hand, the experiments carried out with the catalysts resulted in the value of 12 × 10^–3^, 9.49 × 10^–3^, 12.90 × 10^–3^, 16.83 × 10^–3^, 23.72 × 10^–3^, and 15.81 × 10^–3^ min^–1^, regarding samples β-AgMo, GO@β-AgMo_1, GO@β-AgMo_2.5, GO@β-AgMo_5, GO@β-AgMo_7.5, and GO@β-AgMo_10, respectively as displayed in Table 2.

**Table 2:** Identification, Adsorption (Adsorp.) and Decolorization (Disco.) Percentage, Apparent Constant Rate (*k*_aap_), and Half-Life Time (*t*_1/2_) for All Photocatalytic Experiments

ID	adsorp. (%)	disco. (%)	*k*_aap_ (min) × 10^–3^ min^–1^	*t*_1/2_ (min)
β-AgMo	0.3	58	12.0	57.76
β-AgMo_1	4.1	62	9.49	73.04
β-AgMo_2.5	11.5	73	12.90	53.72
β-AgMo_5	8.2	81	16.83	41.21
β-AgMo_7.5	7.7	90	23.72	29.22
β-AgMo_10	36.5	85	15.81	43.84
photolysis^a^		5	0.38	1824.07
GO	60			
Ag_2_MoO_4_^a^		85.8	9.23	75.0

a Legend: adsorp = adsorption (%); disco. = discolorization; *k*_aap_ = apparent constant rate; and *t*_1/2_ (min) = half-life time. * = Sousa et al.^40^

Based on the data presented in Figure 6a, it was possible to estimate the value of the apparent rate constant (*k*_aap_) of the photodegradation reactions and half-life time (*t*_1/2_) from the −ln plot (*C*/*C*_0_) vs exposure time to UVc light, as shown in Figure 6b and Table 2.

The results summarized in Table 2 confirm the best performance for the GO@-AgMo_7.5 sample, which was approximately 62.5 times faster than that of the test in the absence of the catalyst (photolysis). This makes this catalyst promising for the degradation and consequent discoloration of the RhB dye in an aqueous medium.

When correlating these data with the optical properties of the studied samples, it is noted that they agree with the results obtained for the *E*_VB_ and *E*_CB_ energies, in which the GO@β-AgMo_7.5 sample exhibited a higher energy value for the bands as compared to the other samples obtained in this study, that is, a greater tendency to generate species with oxidizing potential of RhB dye molecules.

**Figure 7:**
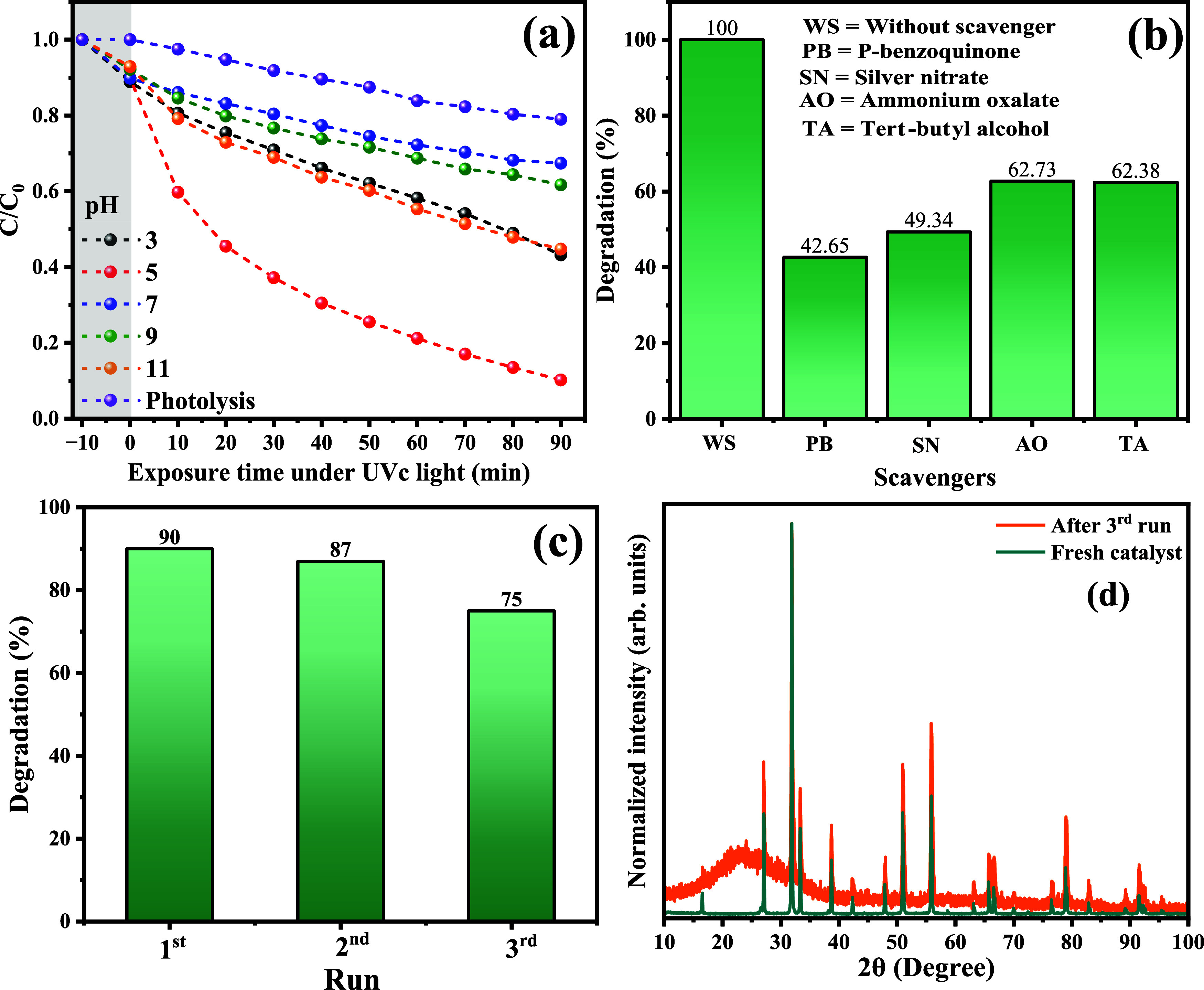
Photocatalytic performance of the GO@β-AgMo_7.5 sample as the catalyst at (a) different initial pH of RhB dye solution, (b) the performance of the scavenger radicals in the photocatalysis of RhB solution, (c) reusability and (d) XRD diffraction pattern of catalyst before (fresh catalyst) and after three consecutive photocatalytic cycles.

In order to investigate the photocatalytic performance of the GO@β-AgMo_7.5 sample under different experimental conditions, photocatalytic tests were carried out by varying the initial pH of the RhB dye solution at values of 1, 3, 5, 7, 9, and 11, and the participation of species in the degradation process of RhB dye molecules through the addition of capturing species was studied, as shown in the graphs presented in Figure 7a,b. Therefore, it was possible to notice a superior performance when the initial pH of the solution was equal to 5, which can be explained by the relationship between the surface charge of the catalyst and RhB dye molecules, which favor the process of photon absorption and the formation of oxidizing radicals. On the other hand, under strongly acidic or basic pHs, the decomposition of silver molybdate into Ag^+^ and MoO_4_^2–^ ions is favorable, while under basic pHs, the formation of silver hydroxide and oxide and metallic silver may occur.

To better investigate the contribution of oxidizing species, the catalytic test was carried out in the presence of superoxide radical scavengers, electrons (e^–^), holes (h^+^), and hydroxyls (HO^•^) using the compounds p-benzoquinone, silver nitrate, ammonium oxalate, and *tert*-butyl alcohol, respectively, as can be seen in Figure 7b. The observed profile confirms the greater reduction in photocatalytic performance when electron capturers (50.66%) and superoxide radicals (57.35%) were added, indicating a greater contribution of these species in the degradation process of RhB molecules. Furthermore, it is possible to suggest that the performance observed in Figure 7a, more precisely, regarding the photocatalytic performance at pH values 3 and 11, can be associated with reducing the silver ions present in the silver molybdate structure. Furthermore, there is possible formation of hydroxyl radicals through the oxidation of HO^–^ ions in solution through the photogenerated holes. Although it was observed that the oxidation of RhB dye molecules suffers attack and consequent breakdown of carbon chains by different oxidizing species, the performance followed the descending order: O_2_^•–^ (57.35%) > e^–^ (50.66%) > h^+^ (37.62%) > OH^•^ (37.27%).

The photochemical stability and reuse performance of the catalyst were investigated in three consecutive cycles of photocatalysis, initially using 100 mL of RhB dye solution at a concentration of 10 mg L^–1^ and 0.1 g of catalyst subjected to exposure to UVc radiation for 90 min under constant magnetic stirring. The results for the performance in the three consecutive cycles of photocatalysis, as well as the diffraction pattern for the catalyst before and after the third cycle, are shown in Figure 7c,d.

As seen in Figure 7c, the performance was reduced by 5% compared to the first photocatalytic cycle. These results demonstrate that this material has satisfactory photocatalytic performance, while no significant decomposition of its structure occurred. This behavior is related to the synergistic effect between GO’s adsorptive profile in the mixture and the photocatalytic performance of silver molybdate, reducing the photo-oxidation processes of silver atoms in silver oxide and other silver-based compounds. Consequently, the crystalline structure and its structural properties were maintained, as confirmed in the diffraction patterns collected before and after the catalytic study, as shown in Figure 7d. The maximum region observed in the 2θ interval between 10 and 40° is due to the profile of the glass substrate used to acquire the diffraction pattern.

**Figure 8:**
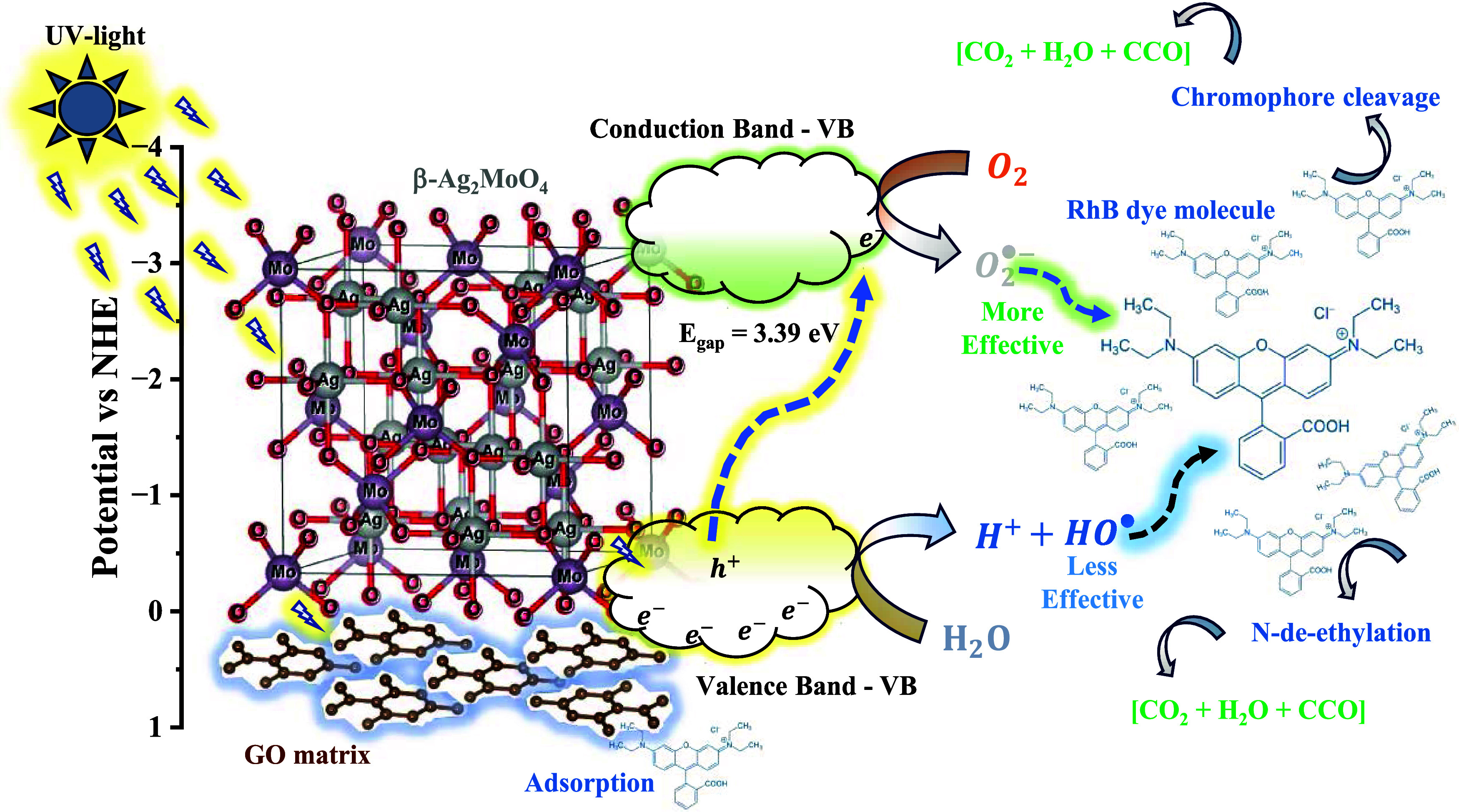
Proposed photocatalytic mechanism of photodegradation for RhB dye by the GO@β-AgMo_7.5 catalyst.

Figure 8 schematically presents the proposed mechanism for the photocatalytic reaction. Initially, the adsorption of the RhB dye molecules occurs on the surface of the catalyst, favored mainly by the GO sheets dispersed on the surface, which have a high adsorption potential and are dispersed on the catalyst’s surface. When absorbing photons with a wavelength equal to or greater than the catalyst bandgap value (*E*_gap_ = 3.39 eV), electrons (e^–^) from the valence band (VB) are excited to the conduction band (CB), resulting in the formation of holes (h^+^) in the VB and the formation of superoxide radicals O_2_^•–^ in the CB after the capture of electrons by the oxygen molecules adsorbed on the catalyst surface. This process is favored by the energy value for the valence (*E*_VB_) and conduction (*E*_CB_) bands, in this case, 0.37 and −3.02 eV, respectively. On the other hand, the formation of h^+^ in VB leads to the formation of H^+^ and OH^–^ species from the oxidation of water molecules, as well as direct oxidation of the carbon chains of the dye molecule. The HO^–^ ions generated from the oxidation of water molecules can also be oxidized to HO^•^ radicals, which also contribute to the attack and breakdown of RhB chains in an aqueous medium. These combined processes lead to the cleavage of the carbon chains of the RhB dye, in addition to N-de-ethylation, resulting in byproducts that, at the end of the process, give rise to gases (CO_2_, N_2_, O_2_) and colorless low-molecular-weight compounds CCO.

## 4 Conclusions

In summary, composites based on reduced graphene oxide (GO) and silver molybdate microcrystals (β-AgMo) were synthesized in different proportions (GO@β-AgMo_1, GO@β-AgMo_2.5, GO@β-AgMo_5, GO@β-AgMo_7.5, and GO@β-AgMo_10) using the hydrothermal method. The combination of materials in different proportions was structurally confirmed by X-ray diffraction (XRD) and Raman spectroscopy, where both materials’ crystallographic planes and vibrational modes were clearly identified. The incorporation of GO in the synthesis of β-Ag_2_MoO_4_ resulted in a reduction in the crystallite size from 79.8 nm (pure) to 71.8 nm GO@β-AgMo_2.5, as well as a reduction in structural micro deformation from 8.40 × 10^–5^ (bare) to −17.2 × 10^–5^ (GO@β-AgMo_2.5). Therefore, there is a compression effect on the structure. The sheet-shaped graphene oxide microstructures and potato-shaped silver molybdate microcrystals were visualized by scanning electron microscopy, confirming the presence of GO distributed on the surfaces of the silver molybdate microcrystals. The photocatalytic tests revealed the best performance for the GO@β-AgMo_7.5 sample, which performed 90% degradation of the RhB dye molecules after 120 min of exposure to UVc radiation. These results are associated mainly with the increase in adsorption performance due to the combination of the materials, as well as the favorable production of superoxide radicals by the photoexcitation of electrons from BV to BC, enabled by the *E*_gap_ value (3.39 eV) and position of the energy band (*E*_CB_ = −3.02 eV), i.e., improvements in the optical properties of silver molybdate.

## Supporting Information Available

The influence of %GO on lattice parameters, a Williamson–Hall plot, a histogram for crystal length distribution by SEM images, the EDS spectrum and UV–vis spectrum of RhB dye solution at different exposure times, and a Table for lattice parameters and Raman active modes band position (PDF)
