# Supplementary material for: GO@β-Ag2MoO4 Composite: One-Step Synthesis, Characterization, and Photocatalytic Performance against RhB Dye
Source: ACS Phys Chem Au. 2024 Sep 10;4(6):632–46. doi: 10.1021/acsphyschemau.4c00038 (PMC11613341; doi:10.1021/acsphyschemau.4c00038)
Supplement: Supplementary file 1 — pg4c00038_si_001.pdf [file pg4c00038_si_001.pdf]

GO@β-Ag_2MoO_4 composite: One-step synthesis, characterization, and photocatalytic performance against RhB dye

Pedro Hyug de Almeida da Silva[1], Dalete Araújo de Souza[1], Rubens Lucas de Freitas Filho[2], Ana Paula de Carvalho Teixeira[2], Rochel Montero Lago[2], Walter Ricardo Brito[3], Edgar Alves Araújo Junior[4], Litiko Lopes Takeno[5], Francimauro Sousa Morais[1], José Fábio de Lima Nascimento[6], Yurimiler Leyet Ruiz[7], Libertalamar Brilhalva Saraiva[1], and Francisco Xavier Nobre[1,6*]

[1]*Departamento de Química, Instituto Federal de Educação Ciência e Tecnologia do Amazonas (IFAM), Campus Manaus Centro, 69020-120, Manaus-AM, Brazil.*

[2]*Departamento de Química, ICEX, Universidade Federal de Minas Gerais, 31270-901, Belo Horizonte-MG, Brazil.*

[3]*LABEL - Laboratory of Bioelectronic and Electroanalytic, Central Analytical Lab, Federal University of Amazonas (UFAM),69077-000, Manaus-AM, Brazil.*

[4]*Laboratório Interdisciplinar de Materiais Avançados (LIMAV), Universidade Federal do Piauí (UFPI), 64049-550, Teresina-PI, Brazil.*

[5]*Departamento Acadêmico de Infraestrutura – DAINFRA, Instituto Federal de Educação Ciência e Tecnologia do Amazonas (IFAM), Campus Manaus Centro, 69020-120, Manaus-AM, Brazil.*

[6]*Laboratório de Ensaios Mecânicos, Automação e Simulação (LEMAS), Polo de Inovação (INOVA) Instituto Federal de Educação Ciência e Tecnologia do Amazonas (IFAM), 69075-351, Manaus-AM, Brazil.*

[7]Laboratório de Processamento de Materiais (LPMat), Centro de Ciências Exatas, Universidade Federal do Amazonas, 69077-000, Manaus-AM, Brazil.

**Corresponding author*: Francisco X. Nobre (francisco.nobre@ifam.edu.br)

Supporting Information

The lattice parameters obtained through structural refinement using the Rietveld method as shown in Figure S1, revealed that for proportions of graphene oxide and silver molybdate of 1 and 2.5% (m/m), there was an increase in the length of the axes of crystallographic coordinates, resulting, consequently, in an increase in the volume of the unit cell. Therefore, it is suggested that in these proportions, there was an effect of self-assembly and direction of the crystalline domains, resulting from the increase in the volume of the unit cell. However, for the proportions of 5, 7.5 and 10%, there was a reduction in these values compared to the values obtained for the proportions of 1 and 2.5%, due to the reduction in the matter transfer process, caused by the oxide particles. graphene in the reaction medium.

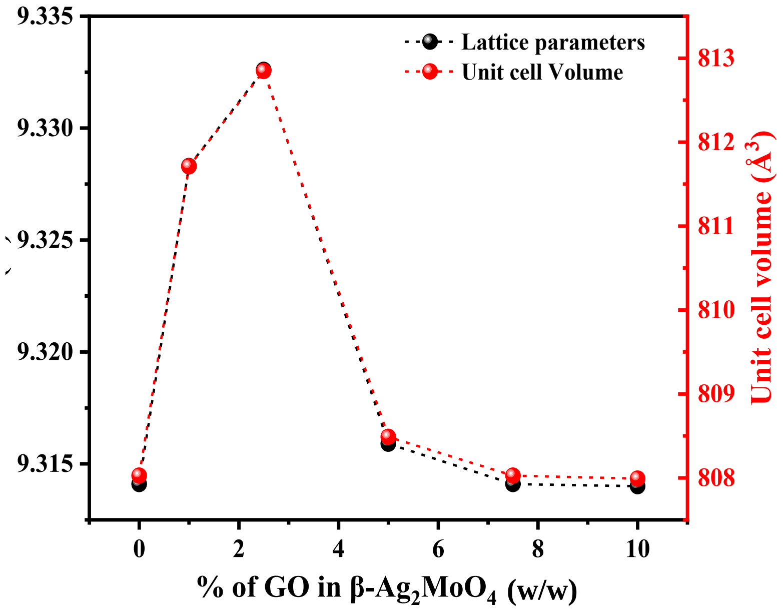

Figure S1: Lattice parameters ($a = b = c$) for and unit cell volume for cubic β-Ag_2MoO_4 for each composite obtained.

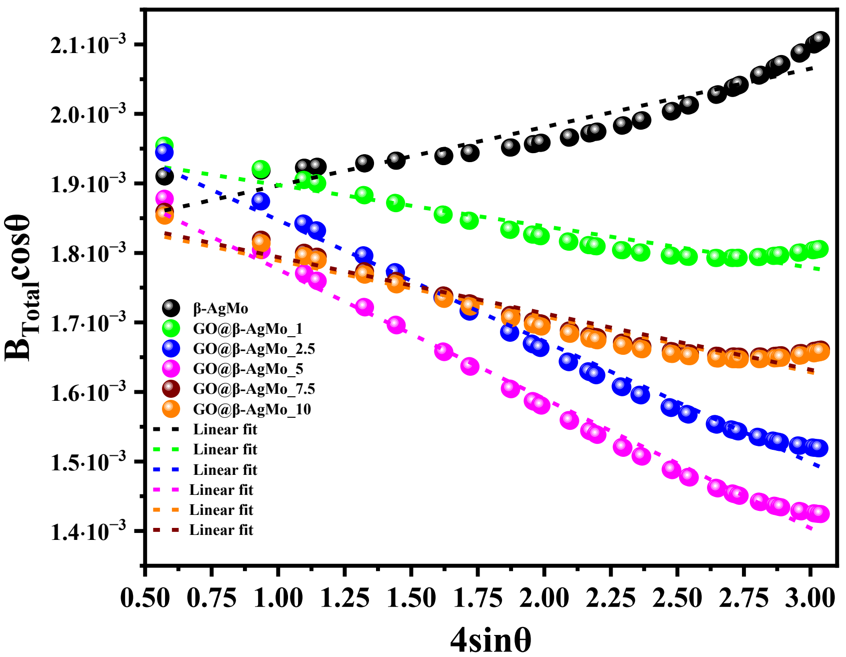

Figure S2: Williamson-Hall plot for β-AgMo and composites (GO@β-AgMo_1, GO@β-AgMo_2.5, GO@β-AgMo_5, GO@β-AgMo_7.5 and GO@β-AgMo_10).

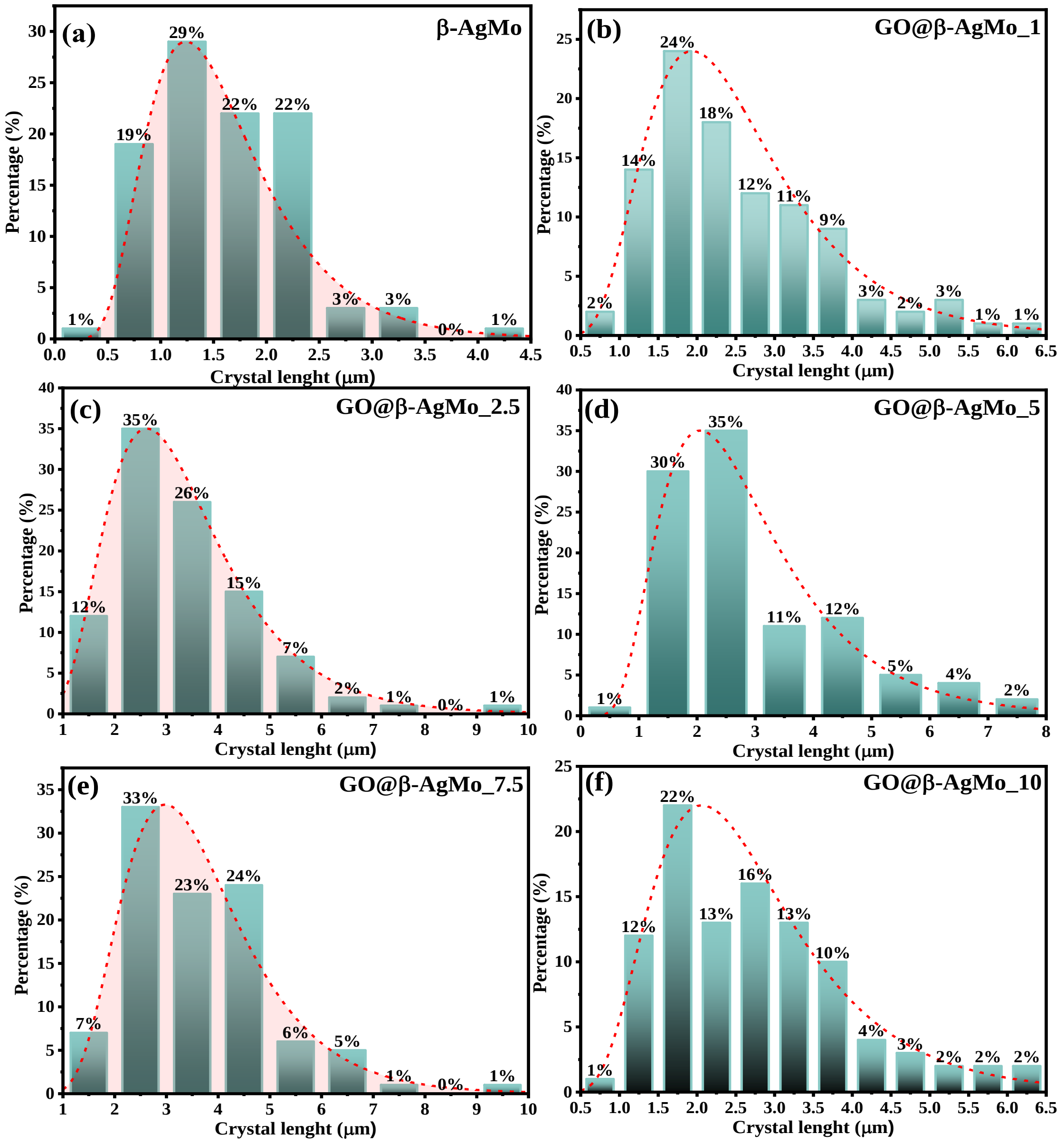

Figure S3: Crystal length distribution for β-AgMo and composites (GO@β-AgMo_1, GO@β-AgMo_2.5, GO@β-AgMo_5, GO@β-AgMo_7.5 and GO@β-AgMo_10).

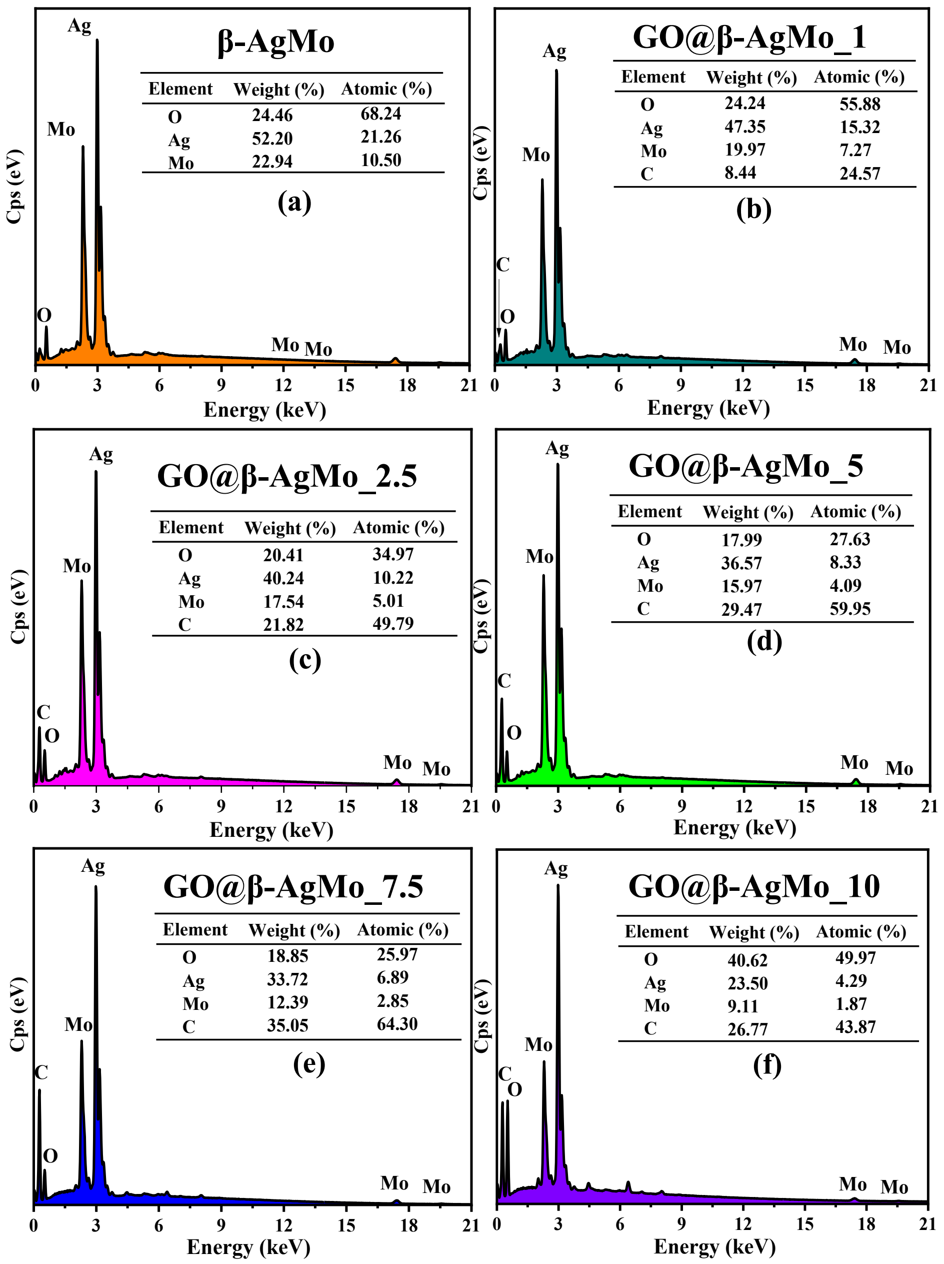

Figure S4: Energy dispersive X-ray (EDX) collected for β-AgMo and composites (GO@β-AgMo_1, GO@β-AgMo_2.5, GO@β-AgMo_5, GO@β-AgMo_7.5 and GO@β-AgMo_10).

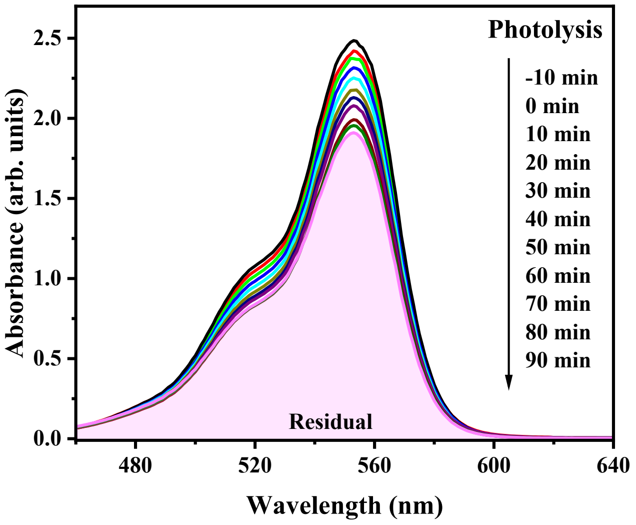

Figure S5: UV-vis spectrum of RhB dye solution at different exposure time under UVc light (photolysis).

Table S1: Rietveld refinement results for atom, Wyckoff, atomic coordinates (*x*, *y*, and *z*), isotropic thermal factor (U_{iso}), and occupancy (O_{cc}) for all synthesized samples, and contained in the ICSD card n° 7222296.

Atom	Wyckoff	Atomic coordinates			U_{iso}	O_{cc}
		x	***y***	***z***		
β-AgMo						
Ag	16d	0.12500	0.12500	0.12500	0	0.94532
Mo	8a	0.5	0.5	0.5	0.16128	2.41838
O	32e	0.23290	0.23290	0.23290	0.16981	4.35454
R_parameters: R_p = 16.3; R_{wp} = 20.1; R_e = 14.1 and χ^2 = 2.03.						
**Atom**	**Wyckoff**	**Atomic coordinates**			**U_{iso}**	**O_{cc}**
		x	***y***	***z***		
GO@β-AgMo_1						
Ag	16d	0.12500	0.12500	0.12500	0	0.66511
Mo	8a	0.5	0.5	0.5	0.69575	1.75561
O	32e	0.23098	0.23098	0.23098	0.46763	3.21298
R_parameters: R_p = 15.9; R_{wp} = 18.8; R_e = 11.1 and χ^2 = 2.84.						
**Atom**	**Wyckoff**	**Atomic coordinates**			**U_{iso}**	**O_{cc}**
		x	***y***	***z***		
GO@β-AgMo_2.5						
Ag	16d	0.12500	0.12500	0.12500	0	0.66265
Mo	8a	0.5	0.5	0.5	0.69575	1.75575
O	32e	0.23215	0.23215	0.23215	0.46763	3.21650
R_parameters: R_p = 17.9; R_{wp} = 20.7; R_e = 12.7 and χ^2 = 2.64.						
**Atom**	**Wyckoff**	**Atomic coordinates**			**U_{iso}**	**O_{cc}**
		x	***y***	***z***		
GO@β-AgMo_5						
Ag	16d	0.12500	0.12500	0.12500	0	0.63043
Mo	8a	0.5	0.5	0.5	0.53019	1.67128
O	32e	0.23224	0.23224	0.23224	0.43174	3.02756
R_parameters: R_p = 13.5; R_{wp} = 17.2; R_e = 11.5 and χ^2 = 2.23.						
**Atom**	**Wyckoff**	**Atomic coordinates**			**U_{iso}**	**O_{cc}**
		x	***y***	***z***		
GO@β-AgMo_7.5						
Ag	16d	0.12500	0.12500	0.12500	0	0.63122
Mo	8a	0.5	0.5	0.5	0.47913	1.64652
O	32e	0.23196	0.23196	0.23196	0.34004	2.89927
R_parameters: R_p = 11.6; R_{wp} = 15.6; R_e = 9.50 and χ^2 = 2.69.						
**Atom**	**Wyckoff**	**Atomic coordinates**			**U_{iso}**	**O_{cc}**
		x	***y***	***z***		
GO@β-AgMo_10						
Ag	16d	0.12500	0.12500	0.12500	0	0.63268
Mo	8a	0.5	0.5	0.5	0.29312	1.64201
O	32e	0.23304	0.23304	0.23304	0.03751	2.86663
R_parameters: R_p = 14.9; R_{wp} = 18.1; R_e = 12.5 and χ^2 = 2.10.						
**Atom**	**Wyckoff**	**Atomic coordinates**			**U_{iso}**	**O_{cc}**
		x	***y***	***z***		
COD no. 7222296						
Ag	16d	0.12500	0.12500	0.12500		
Mo	8a	0.5	0.5	0.5		
O	32e	0.23064	0.23064	0.23064		

Legend: O_{cc} = occupation; U_{iso} = Thermic isotropic factor.

Table S2: Raman active modes and band position for all prepared samples and reported by literature.

Modes	Experimental Raman band position of samples (cm^{-1})						Literature	
	0*	1*	2.5*	5*	7.5*	10*	♠ (cm^{-1})	♣ (cm^{-1})
T_{2g}	89	98	96	79	86	86	102	
E_g	273	274	274	273	274	274	296	
T_{2g}	349	349	347	346	349	349	372	
T_{2g}	759	759	760	757	758	758	779	
A_{1g}	871	871	872	871	871	871	894	
D			1347	1349	1340	1340		1325
G			1595	1581	1579	1579		1589

Legend: 0* = β-AgMo; 1* = GO@β-AgMo_1; 2.5* = GO@β-AgMo_2.5; 5* = GO@β-AgMo_5; 7.5* = GO@β-AgMo_7.5; 10* = GO@β-AgMo_10; ♠ = Fabbro et al.[55]; and ♣ = Fesenko et al.[56].
